# The Effect of Withdrawal and Intake of Nicotine on Smokers' Ability to Ignore Distractors in a Number Parity Decision Task

**DOI:** 10.1155/2013/823158

**Published:** 2013-06-16

**Authors:** Stamatina Tsiora, Douglas D. Potter, John S. Kyle, Adele M. Maxwell

**Affiliations:** School of Psychology, University of Dundee, Dundee DD1 4HN, UK

## Abstract

Nicotine's attention enhancing effects are often attributed to enhancement of stimulus filtering by the attention networks. We investigated distractibility in 20 abstinent cigarette smokers (9 hours overnight; phase 1) and tested them again after smoking one cigarette (phase 2). Their performance was compared to 20 nonsmokers (no nicotine intake). In an auditory number parity decision task, participants had to make a forced choice “odd” or “even” decision about centrally presented numbers between 2 and 9, while ignoring laterally presented preceding or simultaneous novel distractors. In phase 1, distractors that preceded goal stimuli slowed reaction times (RTs) more than simultaneously presented distractors in both groups. In phase 2, nicotine intake speeded RTs in smokers in all conditions and reduced RT variability for simple number decisions and simultaneous distractors. Overall, there was a nonsignificant trend for smokers to be less accurate than nonsmokers. Accuracy in the simultaneous distractor condition decreased in both groups in phase 2. We argue that the observed nicotine-induced improvements on behavioral performance primarily reflect enhancement of top-down control of attention.

## 1. Introduction

Nicotine is the main psychoactive agent in tobacco smoke that acts at nicotinic acetylcholine receptors (nAChRs) in the brain and is found to elicit behavioural and physiological effects including the modulation of attention [1], learning, and memory [2], as well as mood states [3]. Evidence from animal and human studies suggests that cholinergic neurotransmission is crucial for attentional processing [4–6]. It is often reported that nicotine-induced improvements in cognitive function are more pronounced in neuropsychiatric disorders that are associated with altered nAChR function such as schizophrenia (both nonsmokers [7] and smokers [8]), ADHD [1, 9], and Alzheimer's disease [10].

Nicotine deprivation in dependent individuals is associated with withdrawal symptoms and pronounced cognitive-attentional deficits that are alleviated after nicotine intake [3]. It has been reported that nicotine withdrawal symptoms emerge as early as 30 minutes after nicotine intake [11], and the structure of associated symptoms as well as their intensity remains constant over time as abstinence levels increase [12]. Nicotine levels and intake are dependent on each individual's metabolic clearance of the drug [13] but also on the route of administration (e.g., ad libitum smoking produces higher levels compared to nicotine gum [14]). The baseline effects of nicotine are often measured in healthy nonsmokers, non-deprived smokers, and sometimes in minimally deprived smokers (up to 2 hours) using transdermal nicotine patches. These include enhanced motor abilities, faster reaction times (RTs), reduced RT variability, and increased response accuracy [2, 15–19].

It has been suggested that nicotine enhances attention function by improving stimulus filtering (of irrelevant distractors) and/or enhancing processing of relevant stimuli [16]. This is consistent with the biased-competition model of attention [20], top-down signals interact with sensory signals facilitating selective perception and action. Recent studies give support, behaviorally and anatomically, for the existence of two distinct attention-orienting networks. The goal-directed (GD) network, controlled by top-down mechanisms, facilitates the cognitive selection of stimuli and responses [21]. The stimulus-driven (SD) network, is strongly influenced by bottom-up processes and responds to events that are not in the current focus of attention. These networks interact during orientation and reorientation of attention indicating an intermediate connection of different neural populations [21–23]. Maintenance and regulation of higher-level actions and thoughts, as well as the coordination of activity across attention networks, are processes of a frontal lobe executive system of which short-term memory is an important component [24, 25].

 Activity in the two orienting networks is frequently studied using paradigms such as the Posner spatial cueing task where targets appear in expected or unexpected locations, or in “oddball” paradigms where targets appear infrequently in temporal sequences. Often, participants are required to discriminate auditory or visual stimuli (GD) while ignoring task-relevant auditory input (SD). Behavioural distraction can also occur when novel stimuli are presented. However, the degree of distraction is found to vary over time according to the perceived informational value of the distractors [26]. For example, in a forced-choice visual RT task [27], where participants had to discriminate between odd and even numbers while presented with preceding irrelevant auditory stimuli (standard tone, deviant tone, or a natural novel sound), RTs for task decisions were prolonged by 17 ms if preceded by novel sounds, whereas deviant tones decreased the hit rate but did not affect RTs. Supporting electrophysiological (EEG) evidence showed that novel sounds elicited an enhanced negative deflection around 100 ms after stimulus onset (N1), suggested to reflect a transient-detector mechanism activated by novelty, overlapping with the mismatch negativity (MMN), reflecting a stimulus-change detector mechanism. These components were followed by a large positive response approximately 300 ms after stimulus onset (P3a) that consisted of an early central (230 ms after stimulus onset) and a late right-frontal component (315 ms after stimulus onset) associated with violation of expectation and orienting of attention, respectively [27]. It has been shown [28] that following distraction, a later negative component termed reorienting negativity (RON) reflects the process of reorienting attention to the primary task. Thus, when stimulus onset asynchrony (SOA) is, for example, 300 ms, there is sufficient time for the attention system to orient to a distractor and return to the primary task with a small cost in RT. On the other hand, when distractor and target onsets are simultaneous, less behavioural distraction is observed [23]. It has been demonstrated that patients with lesions in the superior parietal lobule, which is an area responsible for top-down control of attention, exhibit a pattern of performance termed “hyper capture” [23]. In this case, the evidence suggests that the GD system is not suppressing attention-switching signals from the SD system.

 It is only relatively recently that studies have utilised nicotine in distractor paradigms [16, 29]. Although findings are inconsistent, nicotine is found to reduce response latencies and it has been suggested that this is due to decreased involuntary shifts of attention and to enhanced attention reorienting on task-relevant stimuli after distraction [16]. In the present study, we employed an auditory number decision paradigm as the goal task with two types of distractors (preceding or overlapping with the goal stimulus) in cigarette smokers after overnight abstinence (phase 1) and after smoking of one cigarette (phase 2). Smokers' performance was compared to a control group of nonsmokers who did not have any nicotine. The behavioral endpoint measures were accuracy (% of correct responses), RT and RT variability (as indexed by the coefficient of variation (CV)). We hypothesized that, relative to simple number decisions, distractors preceding goal stimuli would slow RTs, increase response variability, and decrease accuracy. In contrast, we predicted that distractors presented simultaneously with the goal stimulus would distinguish smokers from nonsmokers in the following way. We predicted that abstinent smokers would show evidence of enhanced distractibility (slower RTs, enhanced RT variability, and reduced accuracy) and that nicotine intake would reduce these effects. 

## 2. Methods

### 2.1. Participants

Participants were twenty right-handed nonsmoking subjects (9 males, mean age = 23 years, SD = 2.5, range = 20–26; and 11 females, mean age = 23 years, SD = 3.3, range = 18–31) and twenty cigarette smokers (mean daily nicotine consumption = 12 cigarettes, SD = 2.5), 8 males (mean age = 26 years, SD = 5.4, range = 17–33), and 12 females (mean age = 24 years, SD = 3.6, range = 18–32) matched for age (*t*(38) = −1.29, *P* = .21). Participants self-reported normal hearing and no current or past psychiatric disorders, use of medication or illicit drugs within the previous month, and use of alcohol in excess (>15 units per week). The study was conducted in accordance with the 2008 Declaration of Helsinki [30], and all participants gave written informed consent in accordance with guidelines set by the Research Ethics Committee of Dundee University. Smokers gave verbal and written confirmation of their abstinence from nicotine for the agreed period (9 hrs) upon arrival at the laboratory. Subjects did not receive payment for their participation but they entered in a prize draw for an MP3 player.

Smokers were recruited on the basis that they had been regularly smoking for at least six months prior to the study (*M* = 4 years, SD = 2.4). Nicotine dependence was assessed using the Fagerstrom Test for Nicotine Dependence [31], consisting of six items scored in the range of 0 to 1 or 0 to 3 yielding a total score of 10, with higher scores corresponding to higher levels of nicotine dependence. Smokers' scores on the FTND ranged from 2 to 7 with a median score of 5 (*M* = 4.4, SD = 1.7 for males; and *M* = 4.2, SD = 1.5 for females). Nonsmokers were selected on the basis that they had never smoked and they had a score of 0 at the FTND.

### 2.2. Task

Each trial consisted of a pair of auditory stimuli (75 dB SPL) presented binaurally on headphones, with a SOA of 300 ms. In the “control” condition (c), the presentation of a warning sinusoidal tone (100 ms duration; 1000 Hz; rise and fall time of 10 ms) was followed by the central presentation of a number between 2 and 9 (300 ms duration) that was spoken by an adult female native English voice at constant intensity and intonation. In this condition, participants were required to press a button to indicate whether the presented number was odd or even. To explore the effect of distraction of attention on number decisions, the 100 ms tone was replaced by a 100 ms novel sound (“preceding” distractor condition (p)). The novel sounds were unique in each trial and consisted of recordings of a range of environmental sounds with an abrupt onset. In a further “simultaneous” condition (s), onset of lateralized 100 ms novel sounds was synchronized with onset of the binaural 300 ms number stimuli. A further trial type, the “distractor” condition (d), consisted of a monaural 100 ms tone followed by a lateralized 100 ms novel sound (SOA 300 ms). No response was required in this condition. The probability of a trial including a novel stimulus was 0.25.

 Stimulus presentation and response logging were implemented using E-prime software. The experiment consisted of 600 trials, in total, split over two phases (300 trials in each phase). The four stimulus conditions were distributed pseudorandomly across four blocks and the intertrial interval was 2300 ms.

### 2.3. Procedure

Participants were required to attend one morning session that lasted approximately one hour. All participants had been instructed to abstain from excessive consumption of alcohol and smokers to abstain from nicotine for a period of 9 hours overnight. Testing was scheduled at 9 am the next morning. All participants were requested to sign a form giving their informed consent for participating and smokers to confirm their abstinence from smoking for the agreed period. The experiments took place in a sound attenuated laboratory. Participants listened to numbers (between 2 and 9) presented binaurally on headphones and indicated with a button press whether the number they heard in each trial was odd or even. They were instructed not to respond on trials that did not include a number stimulus and to ignore the lateralized novel stimuli that occurred in 25% of the trials. The experiment was organized in two phases with a break of approximately five minutes before phase 2 where the participants in the experimental condition were able to smoke a cigarette supervised by the experimenter and the participants of the control group were able to have a break (without nicotine). The task took approximately 35 minutes to complete.

### 2.4. Treatment

Smokers were instructed to abstain from nicotine for 9 hours prior to participation. Nicotine administration was scheduled before completing the second half of the experiment and it involved smoking of one cigarette. To keep conditions as natural as possible, participants were asked to smoke the brand they were smoking at the time of the experiment and at their usual puff intensity, at regular intervals. All participants finished their cigarette within the 5-minute smoking interval.

### 2.5. Analysis Design

For statistical analyses, IBM SPSS Statistics (version 19.0) software package was used [32]. Accuracy (% correct), mean RTs, and the CV were subjected to analysis of variance (ANOVA). A mixed, repeated measures design was used with between-group factors of smoker/nonsmoker and within-group factors of experiment phase and stimulus type (control, simultaneous, and preceding). False alarms (FAs) in the distractor only condition were computed separately and subjected to ANOVA. Greenhouse-Geisser corrections were applied (when appropriate) to compensate for sphericity violations. Bonferroni adjustment was used to explore significant effects (*P* < .05). Paired and independent samples *t*-tests were used for exploratory analyses.

## 3. Results

### 3.1. Accuracy

**Figure 1:**
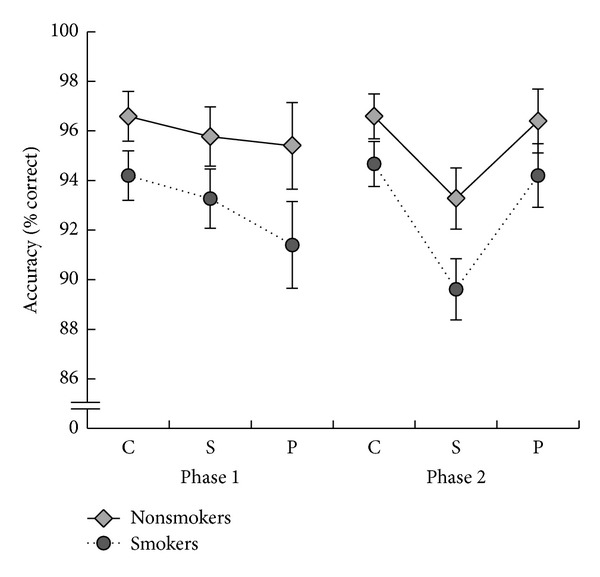
The mean % accuracy and standard error of the mean (SEM) for each stimulus condition (c, s, and p) for smokers and nonsmokers in phases 1 and 2 of the experiment.

Figure 1 illustrates the mean accuracy (% of correct responses) in each of the number decision conditions. In the conditions where a number stimulus was present, a 2 × 2 × 3 mixed repeated measures ANOVA of accuracy (% correct) as dependent measure was carried out with factors of group (nonsmokers, smokers), phase (phase 1, phase 2), and condition (control, simultaneous, and preceding). Mauchly's test of sphericity revealed that the assumption of sphericity was violated for the main effect of condition, *χ* ^2^(2) = 11.94, *P* < .05 and the interaction between phase and condition, *χ* ^2^(2) = 7.15, *P* < .05. Therefore, degrees of freedom were corrected using Greenhouse-Geisser estimates of sphericity (*ε* = .78 for the main effect of condition and *ε* = .85 for the phase × condition interaction). There was a nonsignificant trend for nonsmokers (*M* = 95.67, SE = 1.03) to be more accurate than smokers (*M* = 92.89, SE = 1.03; *F*(1,38) = 3.67, *P* = .063). There was also a main effect of task condition, *F*(1.57,59.57) = 10.40, *P* < .001. However, as can be seen in Figure 1, this is best considered in the context of a significant phase × condition interaction, *F*(1.70,64.64) = 12.52, *P* < .001. Paired samples *t*-tests revealed that in phase 1, accuracy for simple number decisions (*M* = 95, SE = .73) was significantly higher than in the preceding distractor condition (*M* = 93, SE = 1.26; *t*(39) = 2.41, *P* < .05) but not the simultaneous distractor condition. In phase 2, accuracy for simple number decisions (*M* = 96, SE = .65) was significantly higher compared to the simultaneous distractor condition (*M* = 91, SE = .91; *t*(39) = 8.95, *P* < .001) but not the preceding distractor condition.

In the distractor only condition, in which no number was presented and participants should not press a button, ANOVA of FAs as dependent measure revealed only a significant main effect of phase, *F*(1,38) = 5.46, *P* < .05, where participants produced more FAs in phase 1 (*M* = .20, SE = .11) than in phase 2 (*M* = .08, SE = .08).

### 3.2. Reaction Times

**Figure 2:**
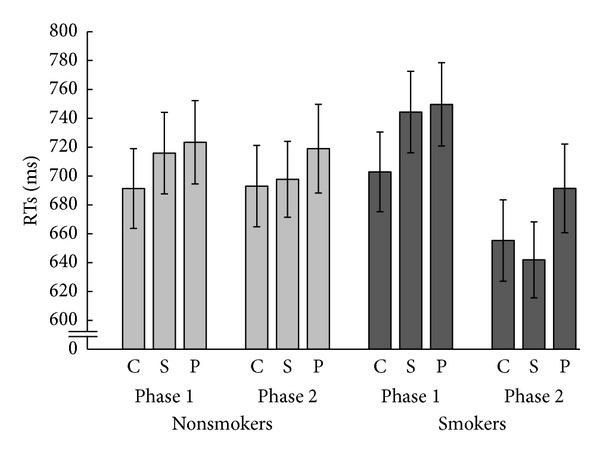
Mean RTs and SEM for each stimulus condition (c, s, and p) for smokers and nonsmokers in phases 1 and 2 of the experiment.

RTs in each of the conditions and groups are illustrated in Figure 2. A 2 × 2 × 3 mixed repeated measures ANOVA of mean RT as dependent measure and factors of group, experiment phase, and condition was carried out. Mauchly's test indicated that the assumption of sphericity had been violated for the main effect of condition (*χ* ^2^(2) = 10.23, *P* < .05) and the phase × condition interaction (*χ* ^2^(2) = 8.48, *P* < .05); therefore, degrees of freedom were corrected using Greenhouse-Geisser estimates of sphericity (*ε* = .81 for the condition and *ε* = .83 for the interaction of phase × condition). There was a main effect of phase, *F*(1,38) = 13.48, *P* < .05, where RTs in phase 2 (*M* = 683, SE = 19.76) were significantly faster than in phase 1 (*M* = 721, SE = 19.48). There was also a significant phase × group interaction, *F*(1,38) = 9.02, *P* < .05. This was due to a significant overall reduction in RTs from phase 1 (*M* = 732, SE = 27.55) to phase 2 (*M* = 663, SE = 27.94) in the smoker group. There was also a significant main effect of task condition, *F*(1.61, 61.21) = 21.95, *P* < .001, and post hoc comparisons revealed that this was due to RTs in the simultaneous (*M* = 700, SE = 18.32, *P* = .004) and preceding distractor conditions (*M* = 721, SE = 10.17, *P* = .000) being significantly slower, overall, than in the simple number parity decision condition (*M* = 686, SE = 18.98). Also, RTs in the preceding distractor condition were significantly slower (*P* = .007) than in the simultaneous distractor condition. However, there was also a significant phase × condition interaction, *F*(1.66,63.08) = 8.51, *P* < .05. Contrasts revealed that compared to RTs for simple number decisions (*M* = 697, SE = 19.49), simultaneous distractors produced a cost of *≅*30 ms (*M* = 730, SE = 19.99) in phase 1 that was significantly reduced in phase 2 (*F*(1,38) = 22.75, *P* = .000; *M* = 674, SE = 19.94 for simple number decisions and *M* = 670, SE = 18.61 for simultaneous distractors).

 Given the large differences in smokers' RTs from phase 1 to phase 2, the scores in each distractor condition (simultaneous, preceding) were subtracted from the scores in the control condition and subjected to a 2 × 2 × 2 mixed repeated measures ANOVA with factors of group, phase, and distractor effect. There was a significant effect of phase, *F*(1,38) = 13.76, *P* < .05 where the effect of distractors was larger in phase 1 (*M* = −36.29, SE = 4.96) compared to phase 2 (*M* = −13.32, *SE* = 4.32). There was also a significant main effect of distractor type, *F*(1,38) = 10.65, *P* < .05 where the effect of simultaneous distractors (*M* = −14.31, SE = 4.09) was significantly smaller compared to the effect of preceding distractors (*M* = −35.23, SE = 5.29). Finally, there was a significant phase × distractor interaction, *F*(1,38) = 6.46, *P* < .05. Paired samples *t*-tests revealed that the effect of simultaneous distractors on RT performance was significantly larger in phase 1 (*M* = −32.99, SE = 5.57) compared to phase 2 (*M* = 4.37, SE = 5.96; *t*(39) = −4.54, *P* < .001) whereas no such change occurred for preceding distractors.

### 3.3. Coefficient of Variation

**Figure 3:**
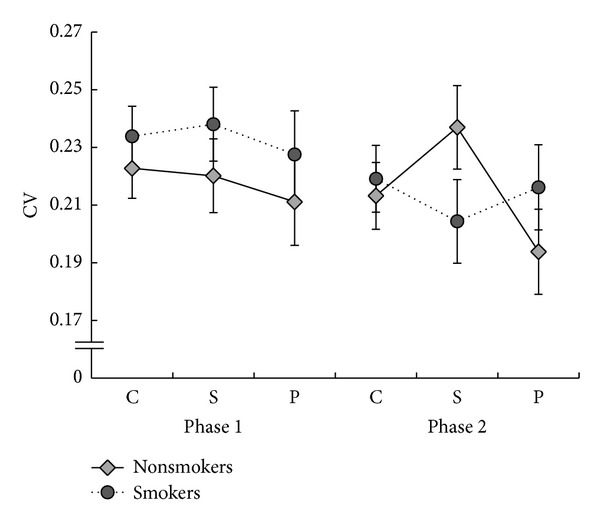
CV and SEM for all conditions (c, s, and p) for nonsmokers and smokers in phases 1 and 2 of the experiment.

The CV was used as a measure of dispersion and was obtained by dividing the SD by the mean RT (see Figure 3).

A 2 × 2 × 3 mixed repeated measures ANOVA on the CV scores revealed a significant main effect of phase, *F*(1,38) = 5.50, *P* < .05, where response variability was significantly higher in phase 1 (*M* = .23, SE = .01) compared to phase 2 (*M* = .21, SE = .01). To explore the magnitude of the distractor effects on RT variability, CV scores in each distractor condition (simultaneous, preceding) were subtracted from the scores in the control condition and subjected to a 2 × 2 × 2 mixed repeated measures ANOVA with factors of group, phase, and distractor effect. There was a significant group × distraction type interaction, *F*(1,38) = 4.13, *P* = .05. However, as can be seen in Figure 3, this is better understood in terms of the nonsignificant trend for a three-way interaction between condition phase and group, *F*(1,38) = 3.05, *P* = .09. Exploratory independent samples *t*-tests revealed that in phase 2, the effect of simultaneous distractors was significantly larger for nonsmokers (*M* = −.02, SE = .01) compared to smokers (*M* = .01, SE = .01; *t*(38) = −2.68, *P* < .05).

## 4. Discussion

This study sought to investigate whether nicotine impacts on the ability to ignore different types of distractors during auditory number parity decisions. We compared performance of a group of cigarette smokers, after they had abstained from smoking for 9 hours and subsequently after smoking one cigarette, to a group of nonsmokers. Nicotine intake produced significant behavioral enhancements, namely, a large reduction in RTs in all conditions and reduction in RT variability when making simple number decisions and when presented simultaneously with distractors. The findings suggest that nicotine improved overall performance by enhancing top-down control of attention. We suggest that these effects possibly also reflect a reduction in involuntary shifts of attention to irrelevant simultaneous distractors. The effect of preceding distractors, which are known to evoke an orienting response, was not, however, reduced indicating no change in the ability to refocus attention on the primary task after distraction.

With regard to the distraction manipulation, the paradigm used was successful in producing decrements in performance in both groups. As previously shown [27, 33], preceding distractors produce performance decrements associated with attention switching to and from the distracting stimulus, whereas no lag between target and distractor onset is associated with smaller decrements in performance [23]. While distraction effects are shown to be greatest when distractors are highly relevant to the task [26], the novel stimuli used in the present study were sufficiently salient to induce reliable distraction effects.

As hypothesized, in phase 1, compared to simple number decisions, preceding distractors and, to a lesser extent, simultaneous distractors slowed RTs in both groups, whereas in phase 2 only preceding distractors significantly slowed RTs. The initial cost of preceding distractors on behavioral performance (phase 1) as measured in the control group was 32 ms. This is consistent with effects observed in other studies where orienting and reorienting responses have been shown to occur in the 300 ms interval between distractor and goal stimulus [28, 33]. The smaller cost of simultaneous distractors on response latencies is consistent with activation of the SD system without subsequent orienting, as a result of modulation of the SD system by the GD system to inhibit bottom-up attentional capture. Overall, RT variability reduced from phase 1 to phase 2 possibly indicating that participants became more accustomed with the task overtime. 

Contrary to our hypothesis that nicotine withdrawal would be associated with impaired performance on the task, we did not find significant differences between groups in phase 1. Given that our participants had their last cigarette before they went to sleep and completed the task within a few hours after waking (9 am), it is possible that this abstinence interval was not sufficient to produce significant effects on performance. However, consistent with our main hypothesis, compared to the control group, smokers' RTs significantly improved in all conditions after smoking a cigarette. Interestingly, the improvement in response speed was emphasized in the simultaneous distractor condition where distraction effects were significantly reduced and performance was analogous to that in the control condition. In addition, simultaneous distractor RT variability in phase 2 was significantly larger for nonsmokers compared to smokers. We believe that nicotine-induced enhancement of top-down control of attention via cholinergic neuromodulation in smokers helped to overcome fatigue during the prolonged demands on attention in this task. Nonsmokers, on the other hand, received no drug and showed increased variability in their responses to simultaneous distractors. Previous research [34, 35] has also found improvements in RTs and intraindividual variability of RT. Attention control is influenced by the level of activation in the Default Mode Network (DMN) which becomes more active during internal thought. Increased DMN activity is associated with increased distractibility but nicotine downregulates activity in the DMN [34]. The changes observed in smokers may therefore be partly due to the effect of nicotine on DMN activation. Finally, contrary to the hypothesized improvement in accuracy after nicotine administration, our analysis indicated a trend for smokers to be less accurate in both phase 1 and phase 2 compared to nonsmokers. 

A number of limitations in this study deserve attention. Firstly, we have no EEG evidence that the distracting stimuli have been processed in the way suggested although previous studies using the same paradigm do show this effect [27]. However, it is evident that distractors impaired performance in the primary task as indicated by RT measures. The failure to test normal hearing, to fully verify smoking abstinence and to verify drug/substance use status is a limitation of this preliminary study. Also, nicotine administration via smoking yields variable nicotine levels and there was no measurement of biochemical indices of absorption. Finally, we did not employ any task performance measure prior to abstinence, which would have been useful to reliably determine the effect of smoking withdrawal on performance. The use of a double-blind, randomized experimental design would have been a better alternative to the design used. Bearing in mind the lack of methodological rigor of the present study, we consider that the lack of differences between groups in phase 1 instills more confidence in the finding that nicotine genuinely improved performance in the second phase of the experiment and it was not only reflecting a reversal of the withdrawal symptoms. Nevertheless, we aim to address the aforementioned issues in future studies. 

## 5. Conclusions

Overnight nicotine withdrawal failed to show significant impairments in performance in a parity decision task that included preceding and simultaneous distractors. However, subsequent nicotine intake via smoking a cigarette significantly reduced response latencies, particularly the effects of simultaneous distractors, and also reduced variability in performance. In contrast, nonsmokers' performance was largely the same in both phases apart from an increase in the coefficient of variability of responses in the simultaneous distractor condition. We suggest that the present preliminary findings demonstrate specific effects of nicotine on cholinergic neuromodulation of top-down control of attention, although further research is needed to determine the robustness of these findings.
